# Advances in brain-heart syndrome: Attention to cardiac complications after ischemic stroke

**DOI:** 10.3389/fnmol.2022.1053478

**Published:** 2022-11-24

**Authors:** Min Wang, Ya Peng

**Affiliations:** ^1^Department of Cardiothoracic Surgery, The Third Affiliated Hospital of Soochow University, Changzhou, Jiangsu, China; ^2^Department of Neurosurgery, The Third Affiliated Hospital of Soochow University, Changzhou, Jiangsu, China

**Keywords:** stroke, brain-heart syndrome, myocardial markers enzymes, cTn, prospects

## Abstract

Neurocardiology is an emerging field that studies the interaction between the brain and the heart, namely the effects of heart injury on the brain and the effects of brain damage on the heart. Acute ischemic stroke has long been known to induce heart damage. Most post-stroke deaths are attributed to nerve damage, and cardiac complications are the second leading cause of death after stroke. In clinical practice, the proper interpretation and optimal treatment for the patients with heart injury complicated by acute ischemic stroke, recently described as stroke-heart syndrome (SHS), are still unclear. Here, We describe a wide range of clinical features and potential mechanisms of cardiac complications after ischemic stroke. Autonomic dysfunction, microvascular dysfunction and coronary ischemia process are interdependent and play an important role in the process of cardiac complications caused by stroke. As a unique comprehensive view, SHS can provide theoretical basis for research and clinical diagnosis and treatment.

## Introduction

Cardiovascular and cerebrovascular diseases are the leading cause of death and disability worldwide and are increasing yearly. According to statistics, from 1990 to 2019, the incidence of stroke was increased by 70%, the prevalence increased by 85%, the mortality rate increased by 43%, and the disability-adjusted life years (DALYs) increased by 32%. The latest statistics for 2019 indicated that ischemic stroke accounts for 62.4% of all strokes (Collaborators, 2021). Ischemic stroke is a common complication of heart disease, and vice versa. Heart injury often leads to adverse events such as early clinical deterioration and death by ischemic stroke (Kumar et al., 2010). As a result, heart injury is the second leading cause of acute death after ischemic stroke and is an important determinant of long-term survival (Scheitz et al., 2015). Currently, treatment of ischemic stroke is dominated by thrombolytic and vascular re-pass therapy (Powers et al., 2018), although most stroke guidelines provide recommendations for post-stroke cardiac examination, including examination for myocardial enzyme profile, routine ECG, 24-h Holter monitoring, and echocardiography (Sposato et al., 2020a). However, these routine tests are the basis for assessment of possible causes of heart injury, while the assessment in turn is a prerequisite for specific treatment. Currently, there are no commonly accepted guidelines on the assessment, treatment, or follow-up on patients with heart injury after stroke from the heart perspective. Therefore, it is of great clinical significance to accurately recognize the occurrence and development of brain-heart syndrome and to investigate the interplay roles between the brain and the heart.

## Overview of brain-heart syndrome

Brain-heart syndrome was firstly reported in 1947 by Byer et al. (1947), when the cerebrovascular disease was found to cause myocardial damage and arrhythmias. Since then, the interaction between the brain and the heart has attracted much attention, and the concept of the brain-heart axis (Manea et al., 2015) has been proposed to emphasize the relationship between nerve dysfunction and heart damage. After more than 50 years of research, the concept of brain-heart syndrome has now been well established. Mayo Clinic defines brain-heart syndrome as ventricular wall movement abnormalities caused by central nervous system diseases that may or may not be accompanied by apical abnormalities. These abnormalities are also evidently not related to any primary diseases that may lead to cardiac abnormalities. The symptoms are mainly described as decreased motor function of the left ventricular wall of the heart, and the symptom gradually restores as the treatment of the primary disease (Mierzewska-Schmidt and Gawecka, 2015). As research progresses, we found that these central nervous system disorders include subarachnoid hemorrhage, epilepsy, ischemic stroke, cerebral hemorrhage, infective meningitis, immune encephalitis, migraine, central sleep apnea syndrome, and traumatic brain injury (Dombrowski and Laskowitz, 2014; Osteraas and Lee, 2017; Scheitz et al., 2018; Sposato et al., 2020b)_._ Recently, the incidence of heart injury due to ischemic stroke has become increasingly significant, and the heart injury complicated by acute ischemic stroke has been described as stroke-heart syndrome (SHS) (Scheitz et al., 2018). Traditionally, SHS refers to cardiac complications that occur after stroke (Scheitz et al., 2021b), including arrhythmias, myocardial damage, and cardiac dysfunction. Researchers have recently summarized cardiac complications after ischemic stroke as 5 categories (Sposato et al., 2020a): (1) ischemic and non-ischemic acute myocardial injuries, manifested by elevated cardiac troponin (cTn), which are often asymptomatic; (2) Acute myocardial infarction (AMI) after stroke; (3) Left ventricular dysfunction, heart failure, and post-stroke Takotsubo syndrome; (4) Sudden cerebral-cardiac death after stroke; (5) ECG changes and arrhythmias, including atrial fibrillation (AF) after strok. The risk of atrial fibrillation after ischemic stroke is often higher, and patients with ischemic stroke are 8 times more likely to experience new atrial fibrillation than non-stroke patients (Sposato et al., 2020b). The concept of atrial fibrillation detected after stroke (AFDAS) (Cerasuolo et al., 2017) was proposed to better distinguish between previous atrial fibrillation and stroke-induced atrial fibrillation, and AFDAS was recently included in the 2020 European Society of Cardiology guidelines for the diagnosis and treatment of atrial fibrillation (Hindricks et al., 2021).

## Clinical manifestations of SHS

The concept of SHS (i.e., cardiac manifestations caused by ischemic stroke) implies that a cardiac function disturbance occurs after the appearance of a neurological deficit, and the cardiac dysfunction occurs in the absence of significant underlying cardiac diseases or disorders. Evidence has shown that the frequency and severity of SHS reach the peak within the first 3 days after the event (Jensen et al., 2012; Kallmunzer et al., 2012) and that most stroke-related cardiac disorders are transient, while some patients show poor prognosis (Faiz et al., 2014; Scheitz et al., 2014; Wrigley et al., 2017). The clinical manifestations of stroke-heart syndrome are continuous, ranging from asymptomatic ECG changes or elevated myocardial markers to deterioration of left ventricular function, malignant arrhythmias, and even myocardial infarction. Furthermore, these symptoms of cardiac dysfunction can occur simultaneously (Fure et al., 2006; Ahn et al., 2016).

## ECG changes

Studies have found that pathological ECG changes may occur in 70 to 90 percent of stroke patients with previous ECG showing Q-wave or left ventricular hypertrophy (Khechinashvili and Asplund, 2002; Hjalmarsson et al., 2012). In a cohort with an unknown prior cardiac disease, ECG changes occur in 91 percent of patients with ischemic stroke. Among all, 32 percent of patients with ischemic stroke showed ECG changes after excluding patients with previous cardiac diseases (Khechinashvili and Asplund, 2002). The most common ECG changes after stroke are complex polarization changes, including Q-T interval prolongation, S-T segment changes, or T-wave widening and inversion (Hjalmarsson et al., 2012; Oppenheimer and Cechetto, 2016). ECG changes are more common in patients with elevated myocardial markers when left ventricular dysfunction occurs. Most of these ECG changes are transient and reach the peak early after stroke. Importantly, changes in ECG after stroke are associated with patient prognosis, and previous studies have shown that prolonged Q-T interval is associated with myocardial injury, severe arrhythmias, and sudden cardiac death after stroke (Soros and Hachinski, 2012). Kallmünzer et al. used an automated arrhythmia detection system to detect ECG changes after stroke, and it was found that 25% of 501 patients experienced severe arrhythmia (139 times) that required urgent clinical evaluation within 72 h of stroke onset. In addition, 58% of these patients had clinically relevant episodes of atrial fibrillation. The remaining patients were detected with ventricular or supraventricular tachycardia, sinus node dysfunction, and second-or third-degree AV block. Rapid arrhythmias that occur after stroke are more common than bradycardiographic changes, and senior age and stroke severity are independent risk factors for these arrhythmias (Kallmunzer et al., 2012). Clinical data support the neurological mechanism for patients with AFDAS (Sposato et al., 2014), and animal studies have shown that left atrial myocardial structural changes occur after stroke in rats, which may be the basis for new atrial fibrillation (Balint et al., 2019). AFDAS has specific risk factors and may have a better prognosis than known atrial fibrillation prior to stroke (Sposato et al., 2018). In addition, AFDAS has a lower incidence rate of cardiovascular comorbidities and structural heart disease and lower CHA2DS2-VASc scores than atrial fibrillation known before stroke (Gonzalez Toledo et al., 2013; Sposato et al., 2018; Yang et al., 2019). However, to date, the mechanisms associated with atrial fibrillation after stroke have not been elucidated, and inflammation and autonomic dysregulation may be so far the best explanation (Sposato et al., 2014). It is critical to clarify this pathophysiological pathway.

## Myocardial markers

Current guidelines recommend measurement of myocardial markers at the time of admission in patients with ischemic stroke (Powers et al., 2018) because timely identification of heart disease in patients with ischemic stroke may improve prognosis. CK-MB is not entirely cardiac specific and may also increase after skeletal muscle injury, renal failure, intramuscular injection, strenuous exercise, and drug action (Chen Z. et al., 2017). Currently, myocardial injury is defined as cTn levels above the 90th percentile (McCarthy et al., 2019), and cTn is considered as a more specific and sensitive biomarker for the detection of heart injury and left ventricular dysfunction than CK-MB. Using the latest highly sensitive analysis, 30 to 60% of stroke patients have elevated cTn, and most of them do not have coronary symptoms (Kubo et al., 2011). In the vast majority of patients with cerebrovascular events, elevated cTn occurs in the absence of typical coronary symptoms (e.g., chest pain, dyspnea), clear ECG ischemic changes (e.g., S-segment elevation), or echocardiography findings (McCarthy et al., 2019). Elevated cTn after stroke is more common in older patients and patients with structural heart disease, such as heart failure and coronary atherosclerotic heart disease (Scheitz et al., 2014). In addition, stroke severity and lesion sites may be associated with elevated cTn, particularly lesions in the right insular anterior cortex associated with acute myocardial injury after stroke (Krause et al., 2017). A recent study reported elevated cTn levels in patients with acute predorsal right island cortex ischemic stroke, which can cause autonomic disorders, sympathetic activation, and myocardial injury (Krause et al., 2017). Many studies have shown that elevated cTn is not only damage to the heart itself, leading to poor prognosis and high mortality of the patients (Wrigley et al., 2017; Fan et al., 2018), but also causes an increased incidence of recurrent macrovascular events, impaired cognitive function, and an increased risk of major adverse cardiac events (von Rennenberg et al., 2019; Broersen et al., 2020a, b; Hellwig et al., 2021). At present, clinical differentiation between acute and chronic myocardial injury is distinguished by continuous measurement of cTn, and acute myocardial injury is manifested by an ascending pattern of cTn levels, while chronic myocardial injury caused by structural heart disease does not have this dynamic pattern (Thygesen et al., 2018; DeFilippis et al., 2019). It has been shown that acute myocardial injury may have a higher short-term mortality rate than chronic myocardial injury (Scheitz et al., 2014), and for this reason, the underlying cause of acute myocardial injury after stroke should be identified in a timely manner to improve prognosis. Although cTn has high specificity in identifying and quantifying myocardial injury, it is not specific for the clinical diagnosis of myocardial infarction or cardiac ischemia. Some studies suggested that elevation of cTn caused by myocardial injury induced stroke can be divided into two types of myocardial infarctions. Type 1 myocardial infarction is associated with myocardial infarction due to acute coronary atherosclerotic thrombosis, and type 2 myocardial infarction is due to a mismatch in oxygen demand/supply caused by pathophysiological mechanisms other than atherosclerotic thrombosis. Significantly different clinical management strategies are required for two different types of myocardial infarction (Scheitz et al., 2021b). So far, there are no clear guidelines on how to distinguish between elevated types of cTn, which need to be evaluated jointly by cardiologists and neurologists. Furthermore, the safety of coronary CTA or cardiovascular MRI in stroke patients is still unclear.

## Cardiac dysfunction

Cardiac complications after stroke are not limited to myocardial injury, but the occurrence of left ventricular dysfunction after stroke is also associated with ischemic stroke. Decreased left ventricular ejection fraction and diastolic dysfunction have been widely reported in all types of strokes, but the lack of knowledge of cardiac baseline status before stroke limits the ability to estimate its true incidence rate (van der Bilt et al., 2014; Lee et al., 2016). Left ventricular dysfunction may be accompanied by elevated cTn or not. However, high cTn is associated with a lower left ventricular ejection fraction, the number of hypokinesia segments, and wall movement abnormalities (Oras et al., 2016). In a study of 1,209 AIS patients, 378 (31%) showed at least mild impairment of left ventricular function. Among these, only about one third of the patients have been pre-diagnosed with congestive heart failure (Siedler et al., 2019). Measurements of NT-proBNP have recently become valuable in the rapid diagnosis of heart failure, and elevated NT-proBNP has been shown to be associated with an increased risk of stroke severity and cardiovascular events, and death after ischemic stroke (Tu et al., 2017; Zhao et al., 2020). Recent meta-analyses have shown that the concentration of NT-proBNP in patients with acute ischemic stroke is significantly increased, but in fact, there is cardiac dysfunction after stroke. NT-proBNP levels rose within 24 h of the onset of acute ischemic stroke and remained high over the six-day study period (Xu et al., 2020). Multivariate analysis showed that the National Institutes of Health stroke score (NIHSS) was one of the strongest independent predictors of elevated NT-proBNP levels (Chen et al., 2012). In addition, elevated NT-ProBNP is the strongest independent predictor of long-term adverse clinical outcomes in patients with acute ischemic stroke (Yang et al., 2018). Recent studies have shown that NT-proBNP is associated with bleeding transformation and poor prognosis in stroke patients treated with intravenous thrombolytic therapy (Zhang et al., 2021). In a preliminary single-center study, 4.9% of AIS patients without pre-existing cardiovascular comorbidities developed heart failure during hospitalization, where only one third of heart failure is caused by acute myocardial infarction after stroke (Micheli et al., 2012). In patients with first-time AIS and no history of heart disease, 3.8 percent of patients were newly diagnosed with an HF event within 1 year, compared with 1.3 percent of stroke-free preference-matched patients (Sposato et al., 2020b). Animal models of cerebral ischemia (occlusion of the middle cerebral artery) can exhibit significant cardiac dysfunction (Ishikawa et al., 2013; Chen J. et al., 2017). Previous studies have found cardiac dysfunction in more than 60% of mice with left cerebral artery occlusion (rather than right), which is associated with left insular injury and increased norepinephrine concentrations (Min et al., 2009). Mouse stroke models induce myocardial dysfunction and injury and are associated with cardiac inflammation and fibrosis, particularly after selective damage to mouse insular cortex related sites (Bieber et al., 2017; Veltkamp et al., 2019). In addition, after 8 weeks of right transient cerebral artery occlusion (rather than left), mice experienced deterioration of left ventricular ejection fraction and an increase in left ventricular volume, which is mediated by chronic autonomic dysfunction and ameliorated by β receptor blockers (Bieber et al., 2017). Thus, acute ischemic stroke may cause cardiac insufficiency, suggesting that clinicians need to detect the NT-proBNP levels while paying attention to cTn during admission and treatment.

In summary, the current clinical data and research results show that there may be a certain causal relationship between cardiac complications after stroke. Cardiac complications caused by acute ischemic stroke, including arrhythmias, myocardial injury (mainly manifested as elevated cTn) and cardiac dysfunction are not directly related, but they can occur independently or simultaneously. Therefore, timely diagnosis and treatment of cardiac symptoms after stroke are essential to improve clinical management and reduce mortality.

## Cardiac complications and prognosis of patients with acute ischemic stroke

Cardiac complications are one of the main challenges in the treatment of acute ischemic stroke, and stroke-heart syndrome is receiving increasing clinical attention. In randomized controlled trials, approximately 20 percent of patients with ischemic stroke reported serious cardiac adverse events mainly within the first 3 days after symptom development, including acute coronary syndrome, heart failure, and arrhythmias (Prosser et al., 2007). SICFAIL studies have found that a significant proportion of the population with ischemic stroke exhibit subclinical and clinical cardiac insufficiency (Heuschmann et al., 2021). Troponin has heart-specific isomers, therefore, many studies have identified troponin as a specific biomarker for myocardial injury after acute ischemic stroke. TRELAS studies have shown that nearly half of patients with ischemic stroke who have troponin above the threshold for the diagnosis of myocardial infarction have no evidence of obstructive coronary artery disease (Scheitz et al., 2014). Studies have shown that senior age, a history of structural and coronary heart diseases, and cardiovascular risk factors, including impaired renal function, are risk factors for elevated troponin after ischemic stroke. Furthermore, stroke-related factors such as stroke severity and stroke location are also associated with elevated troponin (Scheitz et al., 2012; Krause et al., 2017). By applying voxel-based symptom localization methods, lesions in the anterior cortex of the right dorsal insula may contribute more to elevated troponin (Scheitz et al., 2012).

It is controversial whether cardiac complications can affect patient prognosis. It has been found that cardiac troponin only occurs in a small number of patients with acute ischemic stroke, and cardiac troponin does not affect clinical outcomes if other risk factors are considered together (Etgen et al., 2005). Some researchers believe that post-stroke Q-T interval prolongation only independently predicts a poor prognosis in the acute phase, but not a prognosis for the year after stroke (Hjalmarsson et al., 2012). The FIND-AF study, which conducted baseline blood collection in 197 patients with cerebral ischemia without atrial fibrillation and followed up for 1 year, found that only high-sensitivity troponin had an independent predictive effect on vascular events and all-cause mortality among all markers, revealed by multivariate analysis. In the absence of acute myocardial infarction, an initial elevation of myocardial troponin is strongly associated with a poor prognosis both in the acute phase and 1 year after stroke (Stahrenberg et al., 2013). A 2018 meta-analysis found that elevated cTn baseline levels in patients with acute ischemic stroke independently predicted an increase in all-cause mortality, and that measurement of cTn at admission may contribute to early risk stratification of death in these patients (Fan et al., 2018). Elevated high-sensitivity troponin has also been found to be independently associated with an increased risk of death or severe disability after stroke (He et al., 2018). Several studies have also provided consistent evidence that myocardial injury in acute ischemic stroke is associated with poorly functioning prognosis and a more than two-fold increase in mortality (Scheitz et al., 2015; Wrigley et al., 2017). At present, the clinical treatment of ischemic stroke is mainly thrombolytic therapy and stent therapy, and studies have found that, regardless of the treatment, the high-sensitivity troponin level is associated with patient mortality (Sui et al., 2019; Wu et al., 2021). In addition to being strongly associated with mortality, recent studies have shown that elevated levels of hs-cTn in stroke patients are associated with an increased incidence of major cardiovascular events (Scheitz et al., 2020; Broersen et al., 2020b). Moreover, hs-cTn levels are also associated with the severity of cerebral small-vessel disease and impaired cognitive function (Broersen et al., 2020a). PROSCIS-B (Berlin Stroke Event Prospective Cohort Study) showed that in patients with mild to moderate first-time stroke, patients with the highest quartile levels of hs-cTn were 1.8 times more likely to develop cognitive impairment than the patients with the lowest quartile. This association remained within the first 3 years after the stroke event (Broersen et al., 2020a). Recent studies have found that the 5-year recurrence rate of stroke in patients with cardiac complications following ischemic stroke is greater than 50% (Scheitz et al., 2021a; Buckley et al., 2022). Since the 21st century, post-stroke cardiac complications have attracted much attention, and multicenter research has focused on improving clinical diagnosis and prediction models as well as determining the patients that need emergency cardiac intervention treatment. Additionally, the role of underlying cardiac disease and stroke-related factors in the development of post-stroke heart injury has yet to be elucidated. These problems need to be clearly addressed through many visionary, observational, and multicenter studies.

## Advances in the treatment of stroke-heart syndrome

Currently, there are no guidelines on how to assess, treat, or follow up ischemic stroke patients with post-stroke heart injury from a cardiac perspective. However, most stroke guidelines do provide recommendations for post-stroke cardiac testing, including but not limited to routine ECG, 24-h Holter ECG, and echocardiography (Bartsch and Deuschl, 2010; Mochmann et al., 2016). These tests can be done to evaluate myocardial injury for specific treatment. Studies have provided detailed treatment decisions for elevated cTn after stroke (Scheitz et al., 2015; Sposato et al., 2020a), patients with acute coronary syndrome can be treated in a timely manner, and there is evidence that early coronary angiography after stroke is feasible and safe (Mochmann et al., 2016). In addition, clinical treatment with low-molecular-weight heparin may be considered for cancer-associated thrombosis, stroke due to cardiogenic thrombosis, and cardiac injury (Thalin et al., 2016). Although the optimal treatment for neurogenic cardiac syndrome has not been determined, the use of β-blockers, α-blockers, or ACE inhibitors may be considered (Akashi et al., 2008). It is well known that patients with a poor prognosis for heart injury after stroke require careful and continuous cardiac follow-up. Traditional Chinese medicine has a unique effect on the treatment of brain-heart syndrome. A large number of studies show that the buyang huanwu soup, ginseng injection, ginseng wheat injection, ginseng pine nourishing heart capsule, ginseng dome glucose injection, etc. are effective for the treatment of brain-heart syndrome (Li et al., 2016; Liu et al., 2016; Yang et al., 2016; Ji, 2020; Sun et al., 2022), and acupuncture from traditional medicine also has a positive effect on brain-heart syndrome (Ya-jun et al., 2020; Liu et al., 2022). It has been shown that Edaravone is a novel free radical scavenger and antioxidant that is highly effective against a variety of acute cerebrovascular diseases and protects the cardiomyocytes of stroke patients (Jiang et al., 2016). Guangzheng Wu treated 70 patients with brain-heart syndrome treated by alprostadil intravenous therapy, and the results showed that compared with conventional treatment, the level of myocardial injury markers and inflammatory indicators in patients after treatment was significantly reduced, which confirmed that alprostadil could increase coronary blood flow, protect cardiomyocytes and inhibit inflammatory response (Bonnin et al., 2018). Wentao Li et al. have found that butylphthalide injection combined with alprostadil injection can better restore the neurological function of brain-heart syndrome, improve blood supply to the brain, and reduce heart damage, which may be related to the removal of oxidative stress products of heart and brain tissues, protection of vascular endothelial cells, and inhibition of inflammatory factors (Wang et al., 2022). In summary, currently, the medical community does not have a clear and unified diagnostic standard for brain-heart syndrome and the pathogenesis needs to be further improved. The treatment is still empirical symptomatic treatment, and no clear therapeutic target has now been identified. Therefore, exploring complex diagnosis and treatment options is the research direction for the treatment of brain-heart syndrome.

## Advances in the study of the molecular mechanism of the stroke-heart syndrome

**Figure 1:**
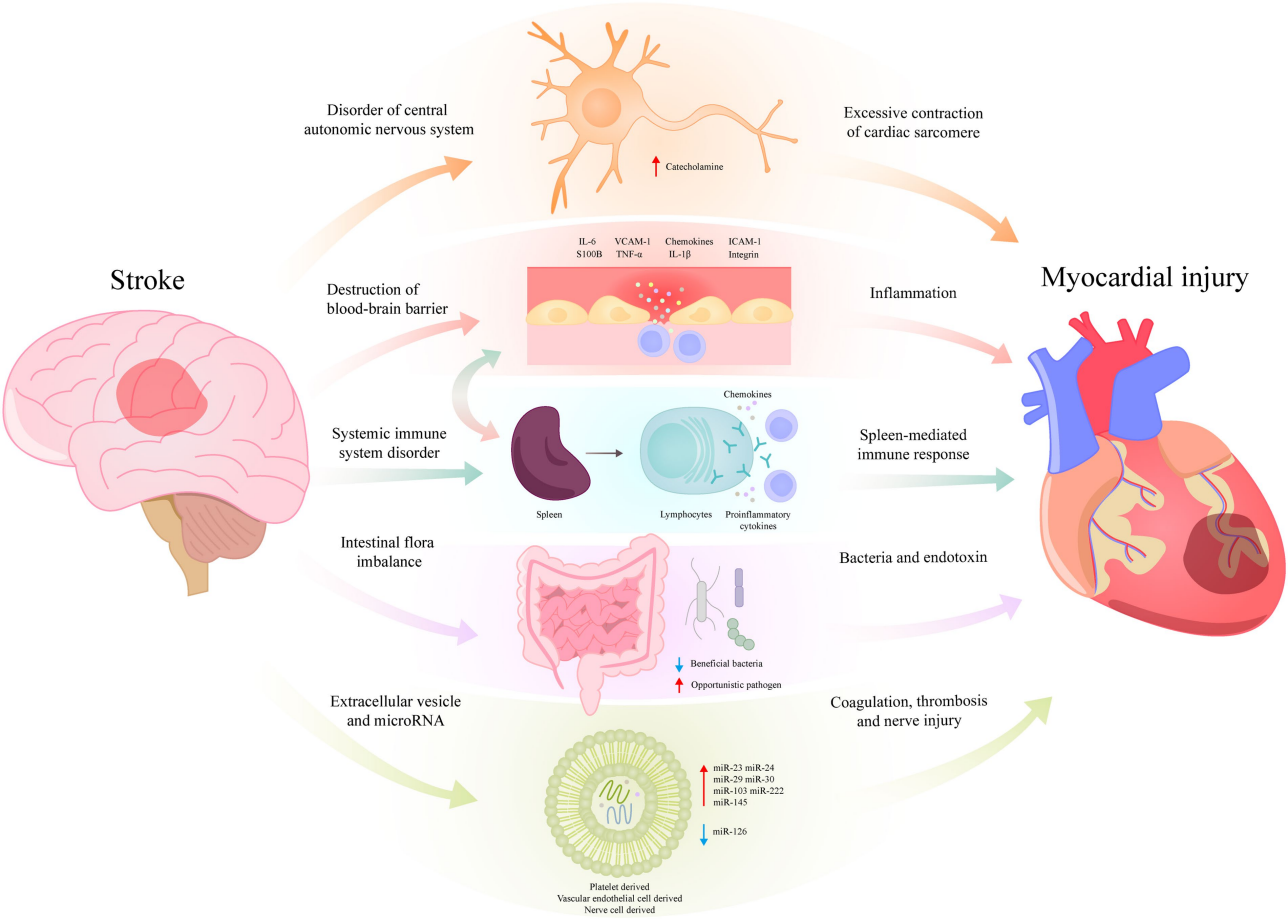
Possible molecular mechanisms of stroke-heart syndrome.

SHS is a complex pathological process, it can occur in patients with or without underlying cardiac disease, in severe cases, it can lead to death. Combined with the evidence of clinical studies and animal experiments, structural and functional disorders of central autonomic neural networks is the currently accepted mechanism of SHS. In addition, the immune response after stroke produced by the spleen or secondary systemic inflammatory response may also be involved in SHS. At present, some studies suggest that intestinal microbiota dysregulation and exosome release after stroke may also be involved in the pathological process of SHS (Figure 1).

## Structural and functional disorders of central autonomic neural networks

Current data strongly supported stroke-induced stress responses to overactivation of central autonomic network structure and dysfunction involving the autonomic nervous system and the hypothalamic–pituitary–adrenal axis. Acute ischemic stroke causes acute lesions of the central autonomic neural network, resulting in acute disturbance of the sympathetic and parasympathetic nerves from the heart. Cardiac sympathetic nerves over-release catecholamines, followed by excessive activation of calcium channels, excessive contraction of cardiac muscle ganglia, weakening of muscle relaxation, and metabolic disorders (Scheitz et al., 2018). One study of 228 patients who underwent MRI scans after ischemic stroke used voxel-based lesion symptom localization to study whether ischemic stroke locally induces myocardial injury. Although a single cTn value is not related to the stroke lesion location, the relative dynamics of this biomarker concentration have a statistically significant correlation with right insula anterior lesions, particularly dorsal subregions (Krause et al., 2017). Furthermore, a study including a consecutive series of 222 patients with ischemic stroke demonstrated that high concentrations of catecholamines were independently associated with myocardial injury following acute ischemic stroke (Ghadri et al., 2018). The most commonly affected area in patients with clinically ischemic stroke is the insular cortex, also known as the site for blood supply to the cerebral artery. Meta-analysis of previous fMRI studies has confirmed that the formation of the insula cortex, prefrontal cortex, cingulate cortex, amygdala, hypothalamus, and hippocampus is an important factor in the central autonomic neural network (Thayer et al., 2012). Similar findings have been observed in animal models. In selectively induced acute ischemic stroke models of insula, the most significant myocardial changes were found around the pulmonary vein near the left atrium, i.e., the area with the highest density of atrial myocardial sympathetic processes (Balint et al., 2019). Additionally, in a mouse model of focal cerebral ischemia for 8 weeks, a phenotype of heart failure with impaired left ventricular ejection fraction and left ventricular dilation was induced in the presence of increased peripheral sympathetic activity (Bieber et al., 2017). Based on this evidence, it seems to explain the pathway of heart injury caused by ischemic stroke from the perspective of autonomic neural networks.

## Destruction of the blood–brain barrier after stroke

The blood–brain barrier consists of endothelial cells, astrocyte endings, pericytes, and the posterior basement membrane, which is located at the core of the neurovascular system. The blood–brain barrier acts as a dynamic interface between the brain and the circulatory system, limiting the entry of potential neurotoxins as well as microscopic and hydrophilic molecules. The destruction of the blood–brain barrier is the main step in the pathophysiological cascade after stroke, and the increased permeability helps inflammatory factors enter the brain, aggravating the ischemia-induced inflammatory response in the brain. Meanwhile, the destruction of the blood–brain barrier causes brain-derived antigens and extracellular vesicles of damaged brain cells to enter the blood and mix with the surrounding immune cells, and ultimately reach the target organ through blood circulation to cause damage to the surrounding tissues (Varatharaj and Galea, 2017). S100B in peripheral blood is a sensitive marker of blood–brain barrier dysfunction and ischemic brain injury and is also a predictor of stroke prognosis (Molnar et al., 2014). Studies have shown that S100B is associated with the lesion area of stroke and the degree of inflammation throughout the body. Furthermore, S100 also plays an important role in cardiomyocyte death and ischemia–reperfusion injury (Frangogiannis, 2019; Li et al., 2019). To date, no studies have shown the role of S100 in heart injury after stroke, but a close relationship between cardiac function and the integrity of the blood–brain barrier has been implied. This effect may be indirect and needs to be elucidated in subsequent studies.

## Systemic inflammatory reaction

The activation of the immune system that causes inflammation after stroke is an important factor in stroke progression. Although cytokines are released acutely after stroke, the increase in plasma levels is only temporary, lasting for a few hours to a few days. Meanwhile, the myocardial remodeling continues despite the short duration of this systemic inflammatory response (Balint et al., 2019). Neuronal cell death after stroke leads to local brain inflammation, and endothelial cells and astrocytes from damaged local brain inflammation are shed into extracellular vesicles and rapidly cross the blood–brain barrier into the bloodstream. When they reach peripheral organs, they release proteins or mRNA to regulate acute cytokine responses and therefore lead to target organ damage (Dickens et al., 2017). Studies have shown that in the initial stages of ischemic stroke attacks, pro-inflammatory cytokines (IL-6, IL-1β, and tumor necrosis factor-α, etc.), integrins, adhesion molecules (ICAM-1 and VCAM-1), and chemokines can further damage the blood–brain barrier and enter the bloodstream system through the blood–brain barrier, inducing a systemic inflammatory response (Yilmaz and Granger, 2010; Liesz et al., 2011). Among them, lymphocytes begin to invade within 48 h after ischemic stroke, and the invading T lymphocytes promote a harmful inflammatory cascade that deteriorates brain damage (Liesz et al., 2011). It has also been found that during ischemic stroke, the spleen plays a central role in the peripheral blood immune response by increasing circulating lymphocytes, pro-inflammatory cytokines and chemokines (Offner et al., 2006; Park et al., 2022). However, splenectomy after 8 weeks of chronic heart failure has been reported to significantly improve left ventricular systolic function and reduce cardiomyocyte hypertrophy (Ismahil et al., 2014). These studies suggest that the spleen-mediated immune response plays an important role in heart damage after stroke.

## Intestinal flora imbalance

As we know more and more about the relationship between the human microbiome and various diseases, it has been found that there may be interactions between the gut microbiota (gut-heart axis) and between the intestinal flora and the central nervous system (brain-gut axis) (Chen Z. et al., 2017). In patients with ischemic stroke, pronounced intestinal dysfunction is manifested by an increase in opportunistic bacteria and a decrease in probiotics (Yin et al., 2015). It has also been suggested that the symbiotic gut flora protects against nerve damage in ischemic stroke, and that their absence or dysregulation increases post-stroke mortality in mice (Winek et al., 2016). In addition, the degree of increased proteus in the intestines of stroke patients is directly proportional to the severity of stroke (Yin et al., 2015). In patients with ischemic stroke, intestinal microbiota disorders and increased bacterial counts of the Lactobacillus rumen subset in the fecal intestinal flora are associated with increased systemic inflammation and metabolic changes (Yamashiro et al., 2017). Systemic inflammation increases intestinal permeability and promotes bacterial and endotoxin translocation to the bloodstream, further triggering a systemic inflammatory response and exacerbating cardiac dysfunction (Nagatomo and Tang, 2015). However, the direct mechanism of action of the intestinal microbiota in the brain-heart syndrome has not been elucidated, and future studies need to focus on the role of the gut microbiome in the brain-heart syndrome and to investigate whether intestinal dysfunction after stroke mediates multi-organ dysfunction of the heart after stroke.

## Circulating microvesicles and microRNA

Many of the circulating microvesicles in the blood come from cells that directly contact the blood, such as platelets and endothelial cells. Elevated circulating microvesicle levels in patients with ischemic stroke are associated with poor clinical prognosis (Huo et al., 2021; Schrick et al., 2022). The main source of microvesicles in stroke patients is vascular endothelial cells, which stimulate endothelial cells to release cytokine IL-6. IL-6 can induce spasm of peripheral blood vessels, including those of the heart, causing coronary syndrome (Schoch et al., 2007). Platelet microvesicles can also serve as a link between vascular coagulation and inflammation in cardiovascular disease, as microvesicles from platelets and damaged brains can lead to changes in platelet activity. Platelet microvesicles have a specific procoagulant activity that is 50–100 times higher than activated platelets. Platelet microvesicles can be found in ischemic stroke followed by thromboembolic events and associated cardiac complications (Sinauridze et al., 2007). Neurons, astrocytes, microglia, and neural stem cells release microvesicles under normal and pathological conditions. Microvesicles can promote the development and progression of certain neurodegenerative and neuroinflammatory diseases (Porro et al., 2015). Previous studies have shown that brain-derived mitochondrial particles are increased significantly in circulation after brain trauma. Mitochondrial particles work synergistically with platelets to promote vascular leakage by disrupting the endothelial barrier. The disruption of the endothelial barrier allows mitochondrial particles to be released into the systemic circulation, promoting coagulation, and enhancing fibrinolysis, vasofibrillin deposition, and thrombosis (Zhao et al., 2016). Therefore, increased coagulation and thrombosis after stroke may cause heart damage and further research is needed for elucidation. MicroRNA (miR) is non-coding RNA and is currently found to be involved in many biological processes, including angiogenesis, inflammation, and hypoxia responses. Some miRs with key cardiac and vascular functions have been reported to be affected after stroke, including upregulated miR-23, miR-24, miR-29, miR-30, miR-103, and miR-222, while miR-126 down-regulated microvesicles are also rich in miRs (Tan et al., 2009). MiR-126 deficiency is highly associated with heart failure, atrial fibrillation, and coronary artery disease, and may be associated with severe stroke-induced cardiac complications (Wang et al., 2008). It has been shown that ischemic stroke reduces the expression of serum and cardiac miR-126 and induces cardiac dysfunction after stroke. Compared with mice in the miR-126 knockout control group, mice with conditionally specific endothelial cell miR-126 knockout exhibit increased cardiac dysfunction after ischemic stroke, and increased myocardial hypertrophy, fibrosis, and expression of inflammatory factors (Chen J. et al., 2017). It has also been shown that circulating miR-145 is also significantly elevated within 24 h of cerebral ischemia, and that circulating miR-145 levels are positively correlated with elevated serum inflammatory factor IL-6 (Dharap et al., 2009). However, whether they are involved in the regulation of cardiac function through inflammatory response needs to be further studied.

## Conclusion

In summary, we describe the broad clinical features and potential mechanisms of cardiac complications after ischemic stroke. Underlying cardiovascular disease and underlying vascular or stroke risk factors increase the incidence of the cardiac syndrome and exacerbate the severity. From the perspective of the mechanism of brain-heart syndrome, autonomic dysfunction, microvascular dysfunction, and coronary ischemic processes are interdependent and play essential roles in the process of cardiac complications caused by stroke. The unique but comprehensive concept of stroke-heart syndrome potentially provides information for clinical decision-making, and further data are needed to provide evidence-based advice on the screening, diagnosis, prevention, and treatment of cardiac complications after stroke.

## Author contributions

MW collected and reviewed the data. YP was responsible for the review and revision. All authors contributed to the article and approved the submitted version.

## Funding

This work was supported by Funding from Young Talent Development plan of Changzhou Health Commission (CZQM2020034 and CZQM2020004), Young talents Science and technology project of Changzhou Health Commission (QN201913), and the National Natural Science Fund (81701584).

## Conflict of interest

The authors declare that the research was conducted in the absence of any commercial or financial relationships that could be construed as a potential conflict of interest.

## Publisher’s note

All claims expressed in this article are solely those of the authors and do not necessarily represent those of their affiliated organizations, or those of the publisher, the editors and the reviewers. Any product that may be evaluated in this article, or claim that may be made by its manufacturer, is not guaranteed or endorsed by the publisher.
